# Validation of the usefulness of artificial neural networks for risk prediction of adverse drug reactions used for individual patients in clinical practice

**DOI:** 10.1371/journal.pone.0236789

**Published:** 2020-07-29

**Authors:** Shungo Imai, Yoh Takekuma, Hitoshi Kashiwagi, Takayuki Miyai, Masaki Kobayashi, Ken Iseki, Mitsuru Sugawara

**Affiliations:** 1 Faculty of Pharmaceutical Sciences, Hokkaido University, Sapporo, Japan; 2 Department of Pharmacy, Hokkaido University Hospital, Sapporo, Japan; 3 Graduate School of Life Science, Hokkaido University, Sapporo, Japan; Vietnam National University, VIET NAM

## Abstract

Artificial neural networks are the main tools for data mining and were inspired by the human brain and nervous system. Studies have demonstrated their usefulness in medicine. However, no studies have used artificial neural networks for the prediction of adverse drug reactions. We aimed to validate the usefulness of artificial neural networks for the prediction of adverse drug reactions and focused on vancomycin -induced nephrotoxicity. For constructing an artificial neural network, a multilayer perceptron algorithm was employed. A 10-fold cross validation method was adopted for evaluating the resultant artificial neural network. In total, 1141 patients who received vancomycin at Hokkaido University Hospital from November 2011 to February 2019 were enrolled. Among these patients, 179 (15.7%) developed vancomycin -induced nephrotoxicity. The top three risk factors of vancomycin -induced nephrotoxicity which are relatively important in the artificial neural networks were average vancomycin trough concentration ≥ 13.0 mg/L and concomitant use of piperacillin–tazobactam and vasopressor drugs. The predictive accuracy of the artificial neural network was 86.3% and that of the multiple logistic regression model (conventional statistical method) was 85.1%. Moreover, area under the receiver operating characteristic curve (AUROC) of the artificial neural network was 0.83. In the 10-fold cross-validation, the accuracy obtained was 86.0% and AUROC was 0.82. The artificial neural network model predicting the vancomycin -induced nephrotoxicity showed good predictive performance. This appears to be the first report of the usefulness of artificial neural networks for an adverse drug reactions risk prediction model.

## Introduction

The process of data mining is defined as the use of techniques to identify hidden correlations and patterns from complex datasets. In addition, it has been described as a method for constructing predictive models based on the discovery of underlying patterns and relationships in large datasets [1].

Artificial neural networks (ANNs) are among the main tools used for data mining. They have a complex computational structure that is inspired by the human brain and nervous system [2]. The structure consists of input and output layers and a hidden layer of units that transform the inputs into something that the output layer can use [3]. ANNs are exceptional tools used for identifying the patterns from complex or numerous datasets to extract and teach the machine to recognise relationships [4–6]. Thus, ANNs are able to incorporate the intricate associations among variables into algorithms. In medical fields, recent studies concerning ANNs have constructed a variety of prediction models: survival prediction of gastric cancer [4], length of stay in an intensive care unit (ICU) [5] and risk of congenital heart disease in pregnant women [6]. Recently, several studies have applied ANNs to investigate adverse drug reactions (ADRs) [7–10]. However, these studies employed ANNs in areas of pharmacovigilance and drug discovery to find a causal relationship between a drug and adverse events [7–10]. Thus, a risk prediction model of ADRs using ANNs that is intended to be used for ‘individual patients in clinical practice’ has not yet been established. Such an ANN would be very useful, so it is important to validate its usefulness when applied to risk prediction models for clinical practice.

In this study, we selected vancomycin (VCM)-induced nephrotoxicity (VIN) for validating the usefulness of ANNs. There are many reports on risk factors for VIN, such as higher concentration (e.g. trough concentrations > 15 or 20 mg/L) [11–13], long-term duration of therapy [14,15], certain hosts (i.e. those with baseline renal impairment and a history of acute kidney injury and those who are critically ill or have septic shock) [16–18] and concomitant medications [i.e. nonsteroidal anti-inflammatory drugs (NSAIDs), furosemide, amphotericin B, aminoglycoside antibiotics and piperacillin–tazobactam (PIPC–TAZ)] [11,19,20]. Thus, risk factors also have been established for the construction of ANNs. In Hokkaido University Hospital, the number of cases of intravenous VCM administration is about 200 patients per year, and this has been estimated to be sufficient for the construction of ANNs [4–6]. Considering the above, VIN was thought to be suitable for verifying the usefulness of an ANN model for the risk prediction of ADR. These risk factors have also been analysed by multiple logistic regression [11–18]. Thus, this conventional statistical method is suitable to validate the ANNs.

Although there are several algorithms for constructing ANNs, we employed a multilayer perceptron (MLP) in this study. MLP is one of the typical supervised learning algorithms in which a small number of parameters can be used to predict outcomes [21,22]. In addition, MLP can be performed by packaging software, such as SPSS (IBM, Tokyo, Japan) and JMP (SAS Institute, Inc., Cary, NC, USA) [4–6,23,24]. Since it does not require complex programming, the methodology established in this research is expected to be easily adaptable to other ADRs by clinicians and pharmacists. Thus, MLP is not new but our approach of applying it to ‘risk prediction of ADR’ is novel.

Therefore, in the present study, our objective was to validate the usefulness of ANNs using MLP algorithm as applied to risk prediction ADRs by constructing a risk prediction model for VIN.

## Materials and methods

### Ethics

This retrospective observational study was conducted in accordance with the guidelines for human studies. The study protocol was approved by the ethics committee of Hokkaido University Hospital (study protocol NO. 018–0379). Because this study is conducted retrospectively, they approved this study and waived informed consent.

### Patients

This single-centre retrospective observational study was conducted at Hokkaido University Hospital. Subjects who had received VCM intravenously from November 2011 to February 2019 were recruited. All data were obtained from the patients’ electronic medical records. The inclusion criteria were (1) age ≥ 18 years, (2) measured VCM trough concentration after the third day of administration and (3) dosing period of ≥3 days. We excluded patients who had undergone haemodialysis and continuous haemodialysis flow or had nephrotoxicity prior to the measurement of VCM trough concentration. Informed consent was obtained from all patients in the form of opt-out on the web-site in Hokkaido University Hospital.

### Criteria of VCM-induced nephrotoxicity

The 2009 vancomycin consensus statement of the Infectious Diseases Society of America [25] has defined nephrotoxicity as a serum creatinine (Scr) increase of ≥0.5 mg/dL or ≥50% relative to baseline [25]. To evaluate VIN, we extracted the maximum Scr during the administration period.

### Data collection

Risk factors for nephrotoxicity were extracted on the basis of previous reports [11–20] and the following potential factors: patient age, sex (male/female), body weight, Scr, creatinine clearance (CCr), duration of therapy, concomitant medications (NSAIDs, furosemide, amphotericin B, aminoglycosides, PIPC–TAZ and vasopressor drugs), residence in the ICU, with or without loading dose and average VCM trough concentration during therapy. Among the concomitant medications, vasopressor drugs were defined as follows: etilefrine, noradrenaline, olprinone, milrinone, dopamine and dobutamine [26]. The loading dose was defined as an initial dose (single or daily) ≥ 1.25 times of the maintenance dosage [26]. Moreover, to evaluate patient characteristics, we collected the days to initial therapeutic drug monitoring (TDM) and initial and maximum VCM trough concentration during therapy. All data were extracted from the beginning of VCM administration, except for the duration of therapy, concomitant medications, residence in ICU, days to initial TDM and VCM trough concentration. Data of concomitant medications and residence in ICU were evaluated during the administration period. To calculate CCr, the Cockcroft–Gault equation was employed [27].

### Construction of the ANN and statistical analysis

As described above, MLP was employed for the construction of ANN. The MLP consists of an input layer of nodes containing information, such as risk factors, followed by a hidden layer of nodes that interact with the input variables that are finally transferred to the output layer [21,28]. In the input layer, the number of neurons depends on the number of independent variables, whereas the number of neurons in the output layer correlates with the number of values that need to be predicted [21,28]. The steps of MLP are summarised as follows [21,28]: (1) data is provided to input layer; (2) input layer produces a predicted output layer, which is subtracted from actual output, and error value is estimated; (3) a back propagation adjusts the weights between output and hidden layer nodes, which works backwards through network; (4) when a back propagation is finished, the process starts again; and (5) this process is repeated until error is minimised.

The analysis was performed in three steps according to previous reports [28]. Firstly, univariate logistic regression analysis was performed to identify the potential risk factors of VIN. All continuous variables were converted into categorical variables. The optimal cut-off points were determined from the receiver operating characteristic (ROC) curves using Youden’s index [29]. Secondly, the ANN and multivariate logistic regression models were constructed. In this analysis, all of the potential risk factors with *P*-values ≤ 0.05 in the univariate analysis were used. Finally, the predictive performances of the ANN model and multivariate logistic regression model were compared. To evaluate predictive performances, the accuracy was calculated for each model, and the areas under the ROC curve (AUROC) of the ANN model was evaluated. These indexes were generally considered to be important performance scores in previous studies [28,30–34]. Furthermore, the 10-fold cross validation was performed for internal validation of the ANN model [24,35]. The Hosmer–Lemeshow test was used to evaluate the fitness of the logistic regression model (the cut-off value was *P* ≥ 0.05) [36].

Patient characteristics were compared using unpaired, and all tests of significance were two-tailed. For comparing the continuous variables, the Mann–Whitney *U*-test was used (all continuous variables were non-normally distributed). Categorical variables were compared using Pearson’s Chi-squared test or Fisher’s exact test. *P* ≤ 0.05 was considered to be statistically significant.

All statistical analyses were performed using JMP 14 (SAS Institute Inc., Cary, NC, USA), a statistical software typically used for ANNs [23,24].

## Results

### Patient characteristics

**Fig 1:**
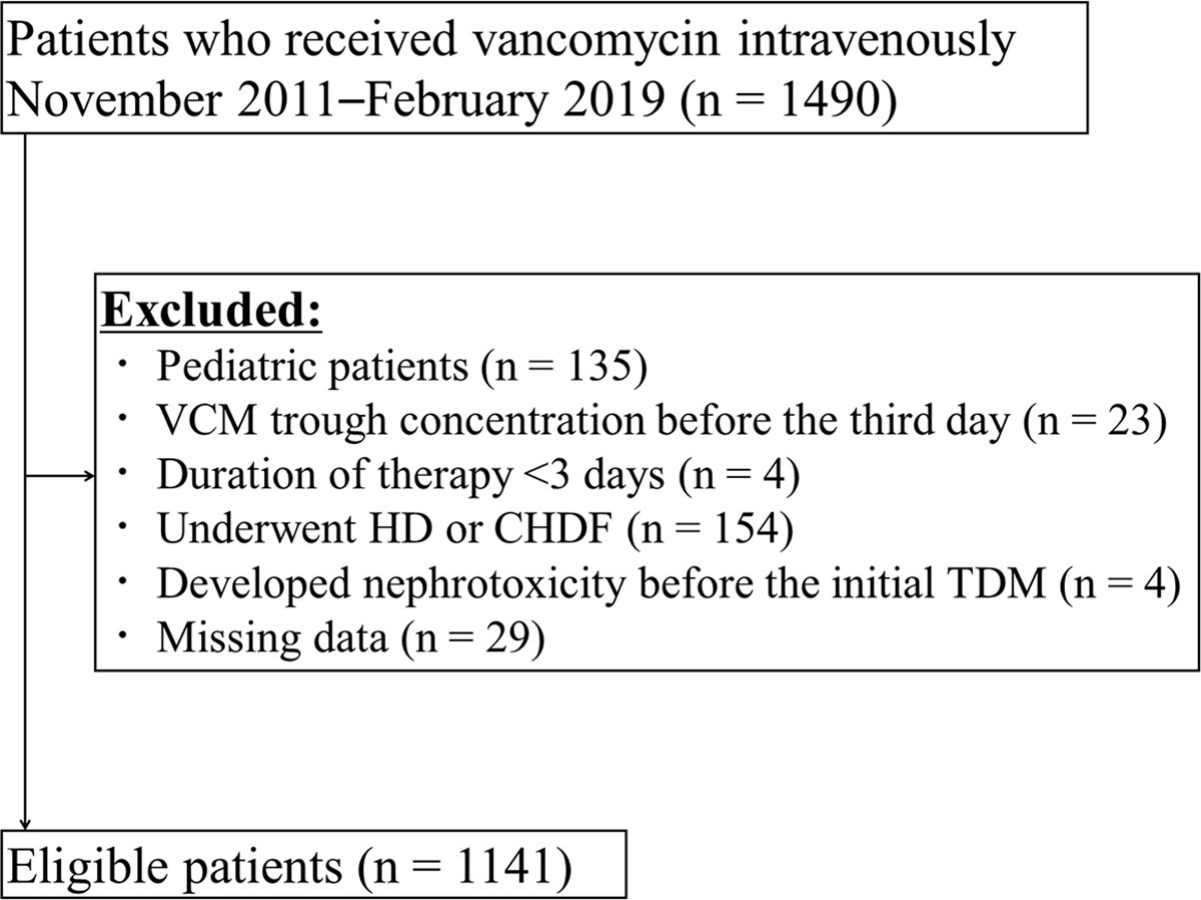
Flowchart of patients included in this study. Vancomycin: VCM, Therapeutic drug monitoring: TDM, Haemodialysis: HD, Continuous haemodialysis flow: CHDF.

Out of 1490 initial patients, 1141 were included in the study (Fig 1). Among them, 179 (15.7%) developed VIN. As shown in Table 1, there were significant differences between the patients who developed nephrotoxicity and those who did not in Scr; CCr; duration of therapy; concomitant medications (furosemide, amphotericin B, PIPC–TAZ and vasopressor drugs); residence in the ICU; and the initial, maximum and average VCM trough concentrations during therapy.

**Table 1:** Comparison of the characteristics of patients with and without nephrotoxicity.

Characteristic	Total (n = 1141)	With nephrotoxicity (n = 179)	Without nephrotoxicity (n = 962)	*P*-value
Age (years), median (range)	65 (18–96)	65 (18–96)	66 (18–95)	*P* = 0.21 ^c)^
Age ≥ 67 years, n (%)	504 (44.2)	72 (40.2)	432 (44.9)	*P* = 0.25 ^a)^
Sex (male), n (%)	728 (63.8)	111 (62.0)	617 (64.1)	*P* = 0.59 ^a)^
Body weight (kg), median (range)	57.0 (28.3–127.0)	57.9 (28.3–98.1)	56.7 (29.1–127.0)	*P* = 0.37 ^c)^
Body weight ≥ 57.2 kg, n (%)	566 (49.6)	99 (55.3)	467 (48.5)	*P* = 0.10 ^a)^
Serum creatinine (mg/dL), median (range)	0.67 (0.16–5.15)	0.62 (0.24–4.57)	0.68 (0.16–5.15)	*P* < 0.01 ^c)^ *
Serum creatinine ≥ 0.68 mg/dL, n (%)	564 (49.4)	71 (39.7)	493 (51.3)	*P* < 0.01 ^a)^ *
CCr (mL/min), median (range)	85.9 (7.3–569.6)	96.3 (7.3–315.2)	84.0 (10.0–569.6)	*P* < 0.01 ^c)^ *
CCr < 88.8 mL/min, n (%)	607 (53.2)	75 (41.9)	532 (55.3)	*P* < 0.01 ^a)^ *
Duration of therapy (days), median (range)	9 (3–88)	12 (3–88)	8 (3–83)	*P* < 0.01 ^c)^ *
Duration of therapy ≥ 10 days, n (%)	533 (46.7)	114 (63.7)	419 (43.6)	*P* < 0.01 ^a)^ *
Concomitant medications, n (%)				
NSAIDs	541 (47.4)	92 (51.4)	449 (46.7)	*P* = 0.24 ^a)^
Furosemide	392 (34.4)	108 (60.3)	284 (29.5)	*P* < 0.01 ^a)^ *
Piperacillin–Tazobactam	188 (16.5)	57 (31.8)	131 (13.6)	*P* < 0.01 ^a)^ *
Amphotericin B	21 (1.84)	11 (6.15)	10 (1.04)	*P* < 0.01 ^b)^ *
Aminoglycoside antibiotics	26 (2.28)	7 (3.91)	19 (1.98)	*P* = 0.17 ^b)^
Vasopressor drugs	149 (13.1)	48 (26.8)	101 (10.5)	*P* < 0.01 ^a)^ *
Residence in intensive care unit, n (%)	145 (12.7)	33 (18.4)	112 (11.6)	*P* = 0.01 ^a)^ *
Duration of initial TDM (days), median (range)	3 (3–10)	3 (3–7)	3 (3–10)	*P* = 0.63 ^c)^
Initial VCM trough concentration (mg/L), median (range)	10.6 (2.1–39.4)	12.8 (3.8–39.4)	10.4 (2.1–36.0)	*P* < 0.01 ^c)^ *
Maximum VCM trough concentration (mg/L), median (range)	13.5 (2.1–72.2)	21.5 (5.7–72.2)	12.6 (2.1–36.0)	*P* < 0.01 ^c)^ *
Average VCM trough concentration (mg/L), median (range)	11.6 (2.1–42.1)	15.1 (4.7–42.1)	11.2 (2.1–29.5)	*P* < 0.01 ^c)^ *
Average VCM trough concentration ≥ 13 mg/L, n (%)	449 (39.4)	114 (63.7)	335 (34.8)	*P* < 0.01 ^a)^ *
With loading dose, n (%)	187 (16.4)	23 (12.8)	164 (17.0)	*P* = 0.16 ^a)^

Creatinine clearance: CCr, Vancomycin: VCM, Nonsteroidal anti-inflammatory drugs: NSAIDs, Therapeutic drug monitoring: TDM

a)Chi-squared test

b)Fisher’s exact test

c)Mann–Whitney *U*-test.

*P-values ≤ 0.05 were considered statistically significant.

### Univariate analysis

In the univariate analysis (Table 2), Scr ≥ 0.68 mg/dL, CCr < 88.8 mL/min, duration of therapy ≥ 10 days, concomitant medications furosemide, amphotericin B, PIPC–TAZ and vasopressor drugs, residence in the ICU and average VCM trough concentration ≥ 13.0 mg/L were significant factors (*P* ≤ 0.05). However, Scr is usually strongly associated with CCr. In this study, Scr was also excluded. Thus, these factors, excluding Scr, were used to construct the ANN and multiple logistic regression models.

**Table 2:** Univariate analysis of risk factors for nephrotoxicity.

Characteristic	OR	95% CI	*P*-value
Age ≥ 67 years	0.83	0.60–1.14	*P* = 0.25
Sex (male)	0.91	0.66–1.27	*P* = 0.57
Body weight ≥ 57.2 kg	1.31	0.95–1.81	*P* = 0.10
Serum creatinine ≥ 0.68 mg/dL	0.63	0.45–0.87	*P* < 0.01^†^
CCr < 88.8 mL/min	0.58	0.42–0.81	*P* < 0.01^†^
Duration of therapy ≥ 10 days	2.27	1.63–3.16	*P* < 0.01^†^
Concomitant medications			
NSAIDs	1.24	0.90–1.70	*P* = 0.19
Furosemide	3.63	2.61–5.05	*P* < 0.01^†^
Amphotericin B	6.23	2.61–14.91	*P* < 0.01^†^
Aminoglycoside antibiotics	1.95	0.69–5.47	*P* = 0.21
Piperacillin–Tazobactam	2.96	2.06–4.27	*P* < 0.01^†^
Vasopressor drugs	3.12	2.12–4.61	*P* < 0.01^†^
Residence in intensive care unit	1.72	1.12–2.63	*P* = 0.01^†^
Average VCM trough concentration ≥ 13 mg/L	3.28	2.35–4.58	*P* < 0.01^†^
With loading dose	0.72	0.45–1.15	*P* = 0.17

Creatinine clearance: CCr, Vancomycin: VCM, Odds ratio: OR, 95% Confidence interval: 95% CI

†*P*-values ≤ 0.05 were included in the artificial neural network and multiple logistic regression analysis.

### Construction of the ANN model

**Fig 2:**
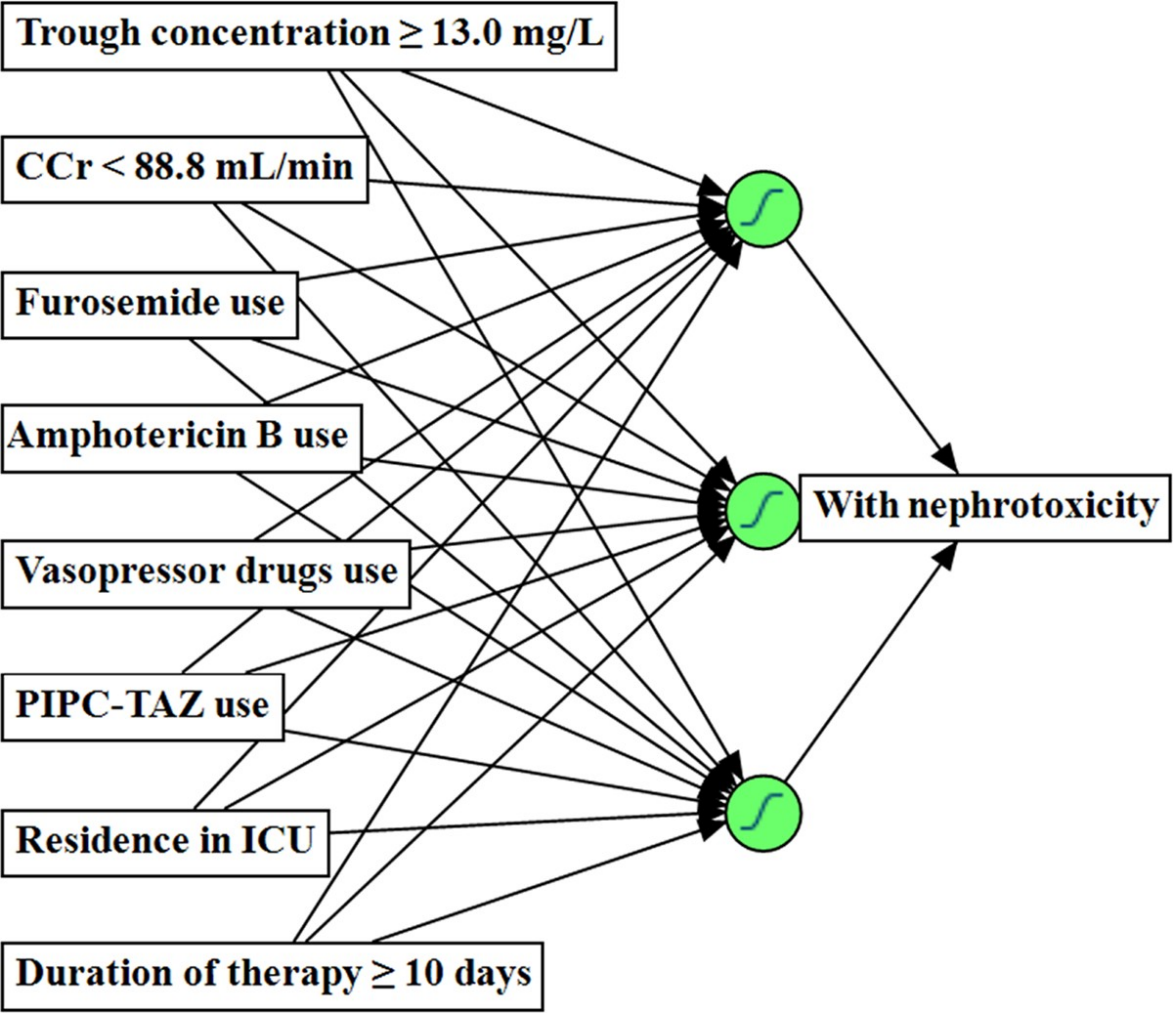
ANN model predicting VCM-induced nephrotoxicity. Creatinine clearance: CCr, Average vancomycin trough concentration: Trough concentration, Intensive care unit: ICU, Piperacillin–Tazobactam: PIPC–TAZ.

**Fig 3:**
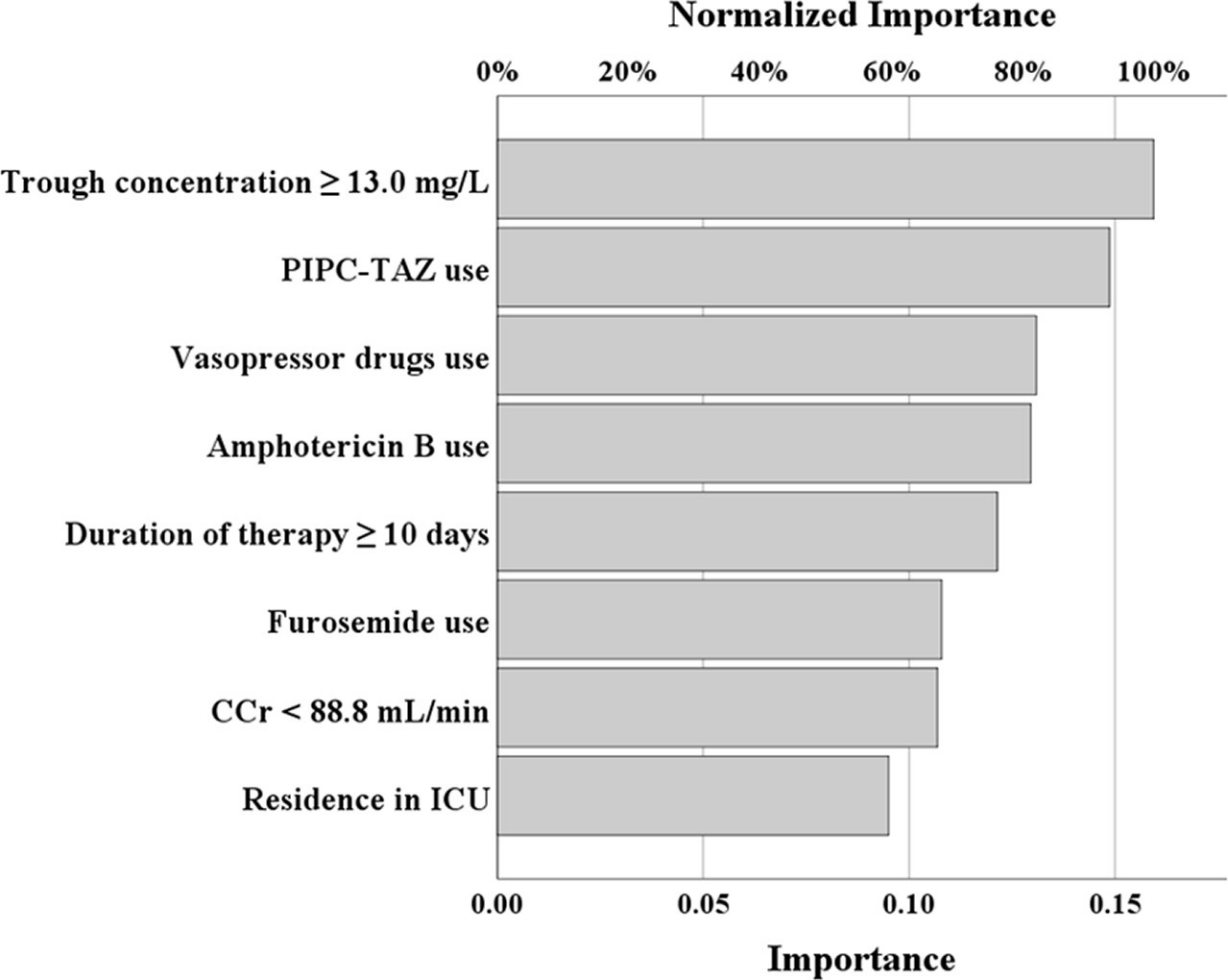
Relative importance of the independent variables in the ANN model. Average vancomycin trough concentration: Trough concentration, Creatinine clearance: CCr, Intensive care unit: ICU, Piperacillin–Tazobactam: PIPC–TAZ.

The ANN model predicting the VIN is shown in Fig 2. Based on the univariate analysis, the eight independent variables were applied, and the dependent variable was the presence or absence of nephrotoxicity. The ANN model consists of an input layer, a hidden layer and an output layer. The input and output layers contained eight and one neuron, respectively. The relative importance of the independent variables in the ANN model is presented in Fig 3. The top three factors for VIN were average VCM trough concentration ≥ 13.0 mg/L, concomitant use of PIPC–TAZ and vasopressor drugs.

### Multiple logistic regression analysis

As shown in Table 3, in the multiple logistic regression analysis using a stepwise approach, CCr < 88.8 mL/min, duration of therapy ≥ 10 days, concomitant medications (furosemide, amphotericin B, PIPC–TAZ and vasopressor drugs) and average VCM trough concentration ≥ 13.0 mg/L were extracted as the independent risk factors of VIN.

**Table 3:** Multivariate analysis of risk factors for nephrotoxicity.

Characteristic	OR	95% CI	*P*-value
CCr < 88.8 mL/min	0.41	0.29–0.60	*P* < 0.01*
Duration of therapy ≥ 10 days	2.32	1.60–3.36	*P* < 0.01*
Concomitant medications			
Furosemide	2.54	1.75–3.68	*P* < 0.01*
Amphotericin B	3.43	1.29–9.11	*P* = 0.01*
Piperacillin–Tazobactam	3.36	2.22–5.06	*P* < 0.01*
Vasopressor drugs	2.78	1.75–4.41	*P* < 0.01*
Average VCM trough concentration ≥ 13 mg/L	3.60	2.49–5.20	*P* < 0.01*

Creatinine clearance: CCr, Vancomycin: VCM, Odds ratio: OR, 95% Confidence interval: 95% CI

*P-values ≤ 0.05 were considered statistically significant.

### Validation of the ANN and multiple logistic regression models

**Fig 4:**
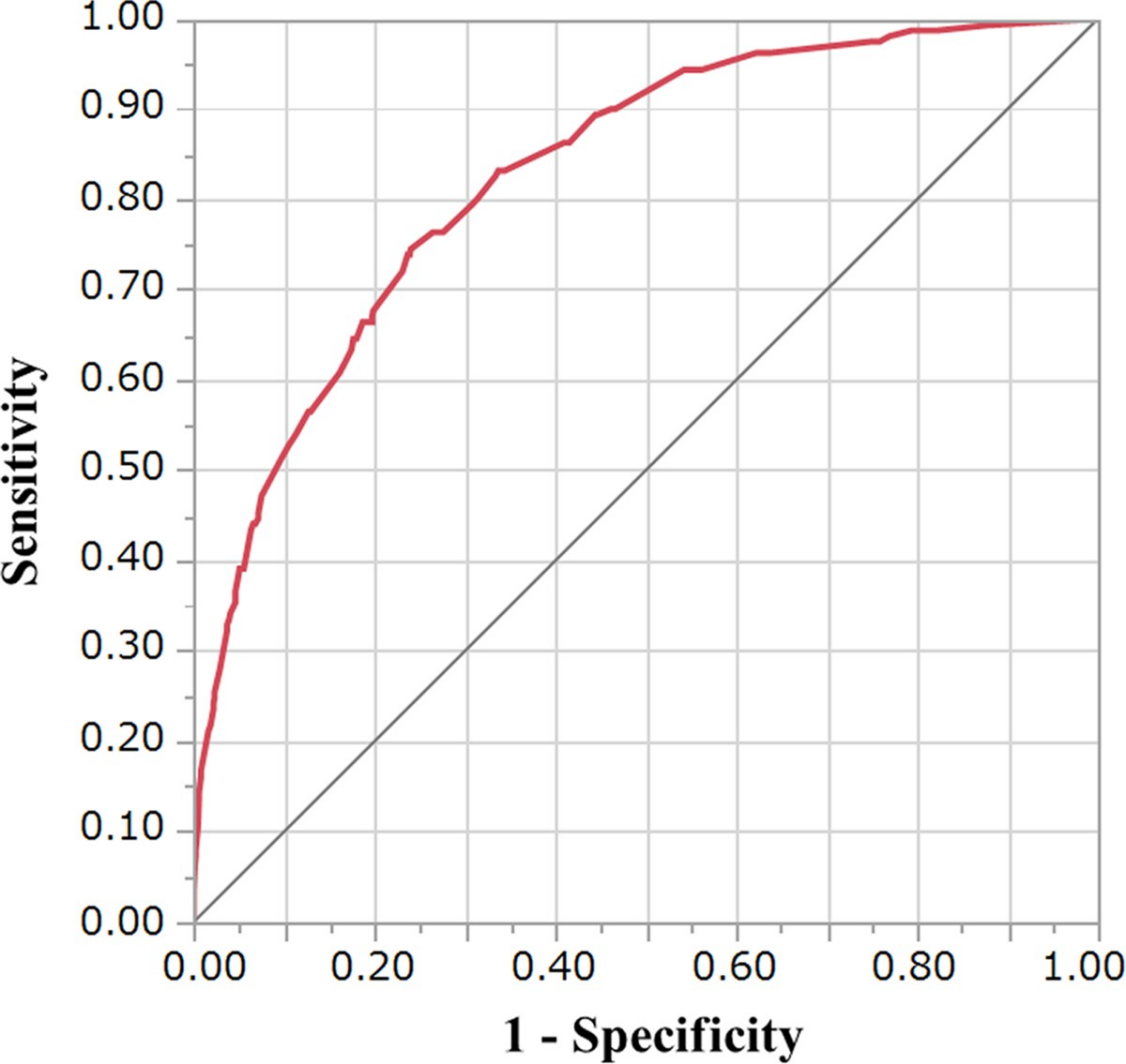
Receiver operating characteristic curve of the ANN model. The area under the receiver operating characteristic curve was 0.83.

The predictive accuracy of the ANN model was 86.3% and that of the multiple logistic regression model (conventional statistical method) was 85.1%. In addition, AUROC of the ANN model was 0.83 (Fig 4). In the 10-fold cross-validation, accuracy and AUROC were 86.0% and 0.82, respectively. In the multiple logistic regression model, the Hosmer–Lemeshow test gave a *P*-value of 0.66.

## Discussion

To the best of our knowledge, this is the first study to validate the usefulness of ANNs applied to a risk prediction model of ADRs for individual patients in clinical practice by constructing a risk prediction model of VIN. In the ANN model, the predictive accuracy was 86.3% and the AUROC was 0.83. These indexes were also used in some previous reports that regarded them as important performance scores [28,30–34]. The AUROC of the ANN model (0.83) indicated moderate accuracy based on the criteria reported by Akobeng [29]. Furthermore, when compared with the results of previous reports, our results are favourable. For example, Pergialiotis et al. built an ANN model to predict endometrial cancer in postmenopausal women and achieved an accuracy of 85.4% [2]. Paydar et al. developed a prediction model of pregnancy outcomes among pregnant women with systemic lupus erythematosus and achieved an accuracy of 90.9% [35]. Hassanipour et al. conducted a systematic review of ten studies that used ANNs to predict health-related outcomes in traumatic patients [30]. They compared AUROC and accuracy between these ten studies, and the AUROC ranged from 0.73 to 0.97, with accuracies from 80.9% to 98.4%. Considering these values, our predictive performances were reasonably accurate. In addition, the accuracy and AUROC in the 10-fold cross-validation was 86.0% and 0.82, respectively, which were favourable [24,35].

In this study, the accuracy of the ANN model (86.3%) was slightly higher than that of the multiple logistic regression model (85.1%). Comparison of the predictive performances of ANNs and logistic regression models has been reported by several previous studies. In the above-mentioned systematic review [30], ANNs had a high level of accuracy and was statistically significant (odds ratio: 1.09). Further, similar results have been obtained in other previous reports [2,31,37,38]. Thus, clinical application of ANNs may enable more accurate prediction of ADRs than logistic regression model. In addition, this approach can be applied to other ADRs and developed further. Meanwhile, logistic regression model is appropriate if the primary endpoint is extracting dependent factors affecting ADRs because ANNs cannot analyse individual factors (e.g., calculating odds ratio) [38].

As shown in Fig 3, an average VCM trough concentration ≥ 13.0 mg/L was extracted as the most important factor of VIN in the ANN, which was consistent with the multiple logistic regression analysis (Table 3). A high VCM trough concentration is known to be a common risk factor of VIN, and cut-off values are usually >15 or 20 mg/L [11–13,16]. On the other hand, our result of ≥13.0 mg/L was lower than these (cut-off points were determined from the ROC using the Youden’s index [29]), which was assumed to be caused by differences in the target trough concentrations. In previous reports, the target trough concentrations were also set to 15–20 mg/L [16,19,25]. In our hospital, target trough levels were set to 10–20 mg/L based on the TDM practice guidelines in Japan [39]. Thus, these target trough levels were lower than those of 15–20 mg/L in previous reports [16,19,25], which may be the reason of the lower cut-off value of VIN. PIPC–TAZ use was extracted as the second most important risk factor in ANN model. Recently, concomitant use of PIPC–TAZ has received attention for its association with VIN [20,40,41]. Although this mechanism remains unclear, VIN is obviously increased by PIPC–TAZ use, and our results supported those of the previous reports. Generally, baseline renal impairment, like that in patients with chronic kidney disease, is associated with VIN [16]. However, our result was inconsistent with this (CCr < 88.8 mL/min, odds ratio = 0.41, 95% confidence interval, 0.29–0.60, Table 3). This is thought to have been caused by the ‘actual Scr use’ in the CCr calculations. Smythe et al. evaluated the accuracy of CCr estimates generated for elderly patients and recommended rounding the Scr to 1.0 mg/dL for low Scr values [42]. In addition, rounding the Scr to 0.6 mg/dL was recommended by Winter [43]. Thus, if an adjusted Scr was employed, this result would not have been obtained. However, an adjustment method for Scr has not become well established, so we used the actual values in the present study. Therefore, investigation of the accuracy of CCr calculations should be investigated in future research.

Accordingly, we also used ANNs to successfully build a risk prediction model of VIN. However, compared with logistic regression analysis, ANNs have several disadvantages. Firstly, ANNs have a ‘black box’ nature; that is, ANNs cannot explain any insights into the structure of the function being approximated [44]. This is in contrast with a logistic regression model that can provide such information. Secondly, ANNs have a risk of overtraining and a possibility of overfitting the model, which may provide an overconfident prediction [45]. Finally, for clinical applications, ANNs require special statistical analysis software. Thus, it would currently be difficult to use our models widely. However, Pergialiotis V et al. explained that these problems can be solved using a larger number of patients (except for requiring the special statistical analysis software) because although a small dataset may not be applicable to large cohorts, the reverse is always possible [2]. Thus, establishment of larger databases, such as one in a multi-centre study, is necessary for the construction of safer ANN models.

Our study had several limitations. First, this study was conducted at a single centre. Second, factors that have been reported previously, such as septic shock, history of acute kidney injury and acute physiology and chronic health evaluation II scores, could not be evaluated [16–18]. In addition, risk factors of concomitant medications and residence in ICU were extracted during the administration period, and trough concentrations were evaluated using average values. Thus, our models included factors that could not be evaluated at the time of use. However, this study aimed to validate ANNs for the prediction of ADRs, so we thought that our study design was the best.

In this study, the ANN model predicting VIN exhibited good predictive performance. Thus, our results indicate the usefulness of ANNs as risk prediction models of ADRs for individual patients in clinical practice. These models would enable clinician and pharmacists to predict ADRs and to easily make decisions such as drug selections. Furthermore, some advanced ANN algorithms, such as recurrent neural network [7,8], can also be employed for this purpose in future. Thus, by performing multi-centre study and using advanced ANN algorithms, reliable risk prediction models need to be built.
